# Predicting Neurobehavioral Outcomes in People with HIV

**DOI:** 10.21203/rs.3.rs-5618870/v1

**Published:** 2025-03-27

**Authors:** Ronald J. Ellis, Bin Tang, Robert K. Heaton, Payal Patel, Jairo Gonzalez, Patricia K. Riggs, Jennifer Iudicello, Scott L. Letendre

**Affiliations:** Department of Neuroscience, University of California San Diego School of Medicine; Department of Psychiatry, University of California San Diego School of Medicine; Department of Psychiatry, University of California San Diego School of Medicine; Department of Neurology, University of Washington School of Medicine; Department of Psychiatry, Icahn School of Medicine at Mount Sinai; Department of Medicine, University of California San Diego School of Medicine; Department of Psychiatry, University of California San Diego School of Medicine; Department of Psychiatry, University of California San Diego School of Medicine

**Keywords:** HIV infection, biopsychosocial phenotypes, machine learning, depression, neurocognitive function, daily functioning

## Abstract

We aimed to identify complex, multidimensional, longitudinal biopsychosocial phenotypes (MLBPSPs) in people with HIV (PWH) and evaluate their associations with baseline clinical characteristics. We included 506 PWH in the multi-site CHARTER study who underwent assessments at four visits, six months apart. Using machine learning, we identified four MLBPSP clusters based on means and nonlinear trajectories of biopsychosocial characteristics. These characteristics included neurocognition, depressed mood, self-reported cognitive symptoms, and activities of daily living at each visit. The largest MLBPSP cluster (C1, N = 231) had the best average scores across all domains and remained stable over 18 months of follow-up. Other clusters showed varying degrees of cognitive impairment, depressed mood, and functional disability. In multivariable analyses, several baseline clinical characteristics, including chronic pulmonary disease, distal neuropathic pain, polypharmacy, and creatinine levels, significantly predicted one or more adverse MLBPSP trajectories. These findings have implications for HIV care by identifying PWH at risk for future adverse trajectories. The results may lead to insights informing future personalized interventions targeted to vulnerable subpopulations of PWH.

## Introduction

Neurobehavioral symptoms and disability have persisted or developed de novo in some people with HIV (PWH) despite viral suppression on antiretroviral therapy (ART). Other PWH are unaffected by these problems; prior cognitive “super-aging” studies have provided partial insight into this subgroup (1–3). Most prior work on neurobehavioral problems has focused on unidimensional characteristics (e.g., isolated neurocognitive [NC] impairment(4, 5) or mood disorders(6–10)), but these often co-occur(11–14) and may have complex, interacting effects. For example, PWH with depressive symptoms may experience increased cognitive and daily functional dificulties (15–17). Relatively little has been done in PWH to address the precision health framework aims regarding individual differences in complex behavioral, psychological, and social features (13, 18), but HIV researchers have only just begun to incorporate these concepts. This has led the field to devise new approaches to understanding complex biopsychosocial (BPS) phenotypes that incorporate a range of neuropsychiatric conditions that can occur in PWH, which might better reflect the biological mechanisms that drive dysfunction(19, 20). Advanced computational methods may identify BPS phenotypes that could reveal biological mechanisms underlying the heterogeneity in this population, leading to clinically useful diagnostic assessments and therapies. These approaches use machine learning to systematically investigate the complex and interrelated factors comprising mood disorders, cognitive deficits, and poor functioning in daily life (19, 21).

In the context of HIV, one investigation applied machine learning to demographic, clinical, medical history, cognitive, and neuroimaging data, finding that psychomotor speed, CD4 + T-cell count, and neuroimaging alterations in motor and visual brain systems collectively differentiated frail from non-frail PWH (22). In another study, machine learning revealed different cognitive profiles by sex (23). Using Kohonen self-organizing maps and 17 neuropsychological metrics, a third report (24) described nine distinct cognitive impairment clusters among virally suppressed female PWH that were related to sequencing, processing speed, learning, delayed recall, and executive functions. Sociodemographic, behavioral, and clinical factors were critical in differentiating between impaired and unimpaired profiles, underscoring this population’s heterogeneity and potentially modifiable factors in cognitive impairment. These studies, however, evaluated only cross-sectional data; BPS phenotypes based on the longitudinal evolution of complex characteristics in PWH have not yet been studied.

The importance of comorbidities and concomitant medications in neurocognitive function has also been highlighted. Many commonly prescribed non-antiretroviral medications have adverse neurobehavioral effects. One study found that medications with anticholinergic properties were associated with worse neurocognitive performance, especially in women with HIV (25). While these factors have been assessed in relation to individual domains such as cognitive impairment and depression, their associations with complex BPS phenotypes are understudied.

To address these gaps in knowledge, we sought to leverage machine learning methods and an innovative, multidimensional approach that is crucial in the context of HIV, where neurobehavioral problems can vary significantly among individuals, even under conditions of viral suppression. By analyzing longitudinal trajectories, we sought to provide deeper insights into the progression of cognitive decline and the long-term effects of HIV on brain function. We aimed to identify biomedical factors at an initial visit that predicted these multidimensional longitudinal BPS phenotypes based on mood, cognitive, and daily functioning features. Biomedical data were selected for their relevance to the modern HIV epidemic and clinical care, such as HIV disease and treatment characteristics, medical comorbidities, concomitant medications, and laboratory tests readily available in the clinic, and BPS features were chosen to address common neurobehavioral problems observed in PWH.

## Methods

### Participants

We studied 506 PWH from the multicenter CNS HIV Anti-Retroviral Therapy Effects Research (CHARTER) cohort enrolled from 2003–2010. Participants had at least two follow-up assessments every six months over approximately 18 months. 28.3% (n = 143) missed measurement of at least one of the 17 MLBPS features. All participants had HIV-1 infection and enrolled without regard to comorbidities to obtain a sample that was as representative as possible of the U.S. population of adult PWH. Exclusion criteria were individuals with active, uncontrolled medical, neurological, or psychiatric conditions that would interfere with study assessments or inability to participate due to ongoing substance use disorder according to DSM-IV criteria within the past 30 days. The local Institutional Review Boards approved the study procedures, and all participants provided written informed consent.

### Biopsychosocial Feature Assessments

We selected BPS assessment tools based on their comprehensiveness and relevance in the context of HIV infection. Additional details on the assessments and analytic approach have been previously published (26). *NC performance* was measured using a comprehensive test battery that assessed seven cognitive domains: verbal fluency, information processing speed, learning, delayed recall, executive function, working memory, and complex motor skills (4). Raw test scores were converted to demographically standardized T-scores (normative mean of 50, SD of 10), which were used to calculate a Global Deficit Score (GDS) as previously described (27, 28). Neuromedical and psychiatric history information was used to classify comorbid neuropsychiatric conditions that could confound the attribution of NCI to HIV according to the published criteria (4, 29). *Current mood symptoms* were evaluated with the Beck Depression Inventory-II (BDI-II), a 21-item, valid self-report instrument (30). Component BDI-II subscales captured cognitive, somatic, affective, and apathy symptoms(31–33). *Daily functioning* was evaluated using the Patient’s Assessment of Own Functioning Inventory (PAOFI)(34) for self-reported NC dificulties in everyday life and an adaptation of the Lawton-Brody instrumental activities of daily living (IADLs) scale (35). PAOFI scores of 3 or above indicate significant experiences of cognitive dificulties in daily life. The IADL instrument assessed possible changes in levels of independence in performing 16 everyday tasks, such as doing laundry and financial management.

### Neuromedical assessments

We selected biomedical assessments for their relevance to the modern HIV epidemic and clinical care, such as HIV disease and treatment characteristics, medical comorbidities, concomitant medications, and laboratory tests readily available in the clinic. Validated comprehensive neuromedical assessments were performed by centrally trained investigators and staff using standardized case report forms described in prior publications(4). ART adherence was assessed using the AIDS Clinical Trials Group method, which considers good adherence as taking at least 95% of doses in the four days before the assessment. HIV infection was diagnosed by enzyme-linked immunosorbent assay with Western blot confirmation. Routine clinical chemistry panels, complete blood counts, hepatitis C virus antibody, and CD4 + T cells (flow cytometry) were performed at each site’s Clinical Laboratory Improvement Amendments (CLIA)–certified laboratory. HIV RNA was quantified by commercial RT-PCR (Amplicor version 1.5, Roche Diagnostics, Indianapolis, IN, USA; lower limit of quantification 50 copies per mL). Nadir CD4 + T-cell count was the lowest self-reported count or study measurement value. The distal sensory polyneuropathy (DSP) and distal neuropathic pain (DNP) evaluations included self-reported neuropathy symptoms (pain, numbness, paresthesias) and a clinical examination for neuropathy signs (bilateral distal vibration, sharp, and touch loss). The Charlson Comorbidity Index (CCI)(36), a summary of the total burden of comorbidities, was calculated for each participant.

### Additional psychiatric and substance use evaluations

Confounding neurocognitive conditions at the baseline visit were judged by experienced HIV clinicians based on published guidance as either incidental (not contributing to NC impairment, e.g., transient headaches), contributing (likely contributing to NC impairment, in addition to HIV itself, e.g., mild depression), or confounding (the principal cause of NC impairment, e.g., severe traumatic brain injury). These ratings showed good clinician interrater agreement in previous studies (37). History of major depressive disorder (MDD) and substance use disorders were assessed using the computer-assisted Composite International Diagnostic Interview (CIDI)(38), a structured instrument widely used in psychiatric research. The CIDI classifies current and lifetime diagnoses of mood and substance use disorders, as well as other mental disorders. Quality of life was assessed using the Medical Outcomes Study HIV Health Survey Short Form 36 (MOS-HIV SF-36), a reliable and valid tool for assessing overall quality of life, daily functioning, and physical health (39). The MOS-HIV contains 36 questions that assess various physical and mental dimensions of health. Items are grouped into two categories (Physical and Mental Health), with 9 subcategories (Physical functioning, Role functioning, Pain, Social functioning, Emotional well-being, Energy/fatigue, Cognitive functioning, General Health, Health distress, and Overall Quality of Life). Higher scores indicate better health.

### Statistical Analysis

A two-step clustering approach was used to identify longitudinal BPS phenotypes in study participants based on 17 features evaluated across four visits (0, 6, 12, and 18 months). The approach was adapted from our previous publication to accommodate longitudinal data (40). Briefly, individual variation in the means and non-linear trajectories of each feature was modeled using linear mixed-effects models (LMMs) with random intercepts and slopes (consisting of a polynomial combination of time: Time + Timê2). Three random effects were fitted: 1) random intercepts; 2) random slopes for the linear effect; 3) random slopes for the quadratic effect, so that 3 new features (1 random intercept and 2 random slopes) were generated for each original feature. For missing data, missing at random was assumed. Using the expectation-maximization (EM) algorithm, LMMs can compute maximum likelihood estimates for data with missing values without requiring imputation[44]. Since the distributions of cognitive DDS, BDI-II subscales, PAOFI components, and IADL total score were all right-skewed distributed, square root transformation was done to improve data normality before fitting an LMM. In the second step, the participant estimates (random effects) were used to cluster participants using k-means. Before clustering, the new 51 features (17 × 3) were reduced to a lower dimension with a principal components analysis. The first 18 principal components (PC1-PC18), explaining approximately 85% of the total variance in the data, were used for cluster analysis. Using the elbow method (based on within-clusters sum of squared errors for the different number of clusters, e.g., from k = 1 to 9), the optimal number of clusters was determined in the k-means clustering. To assess the effect of plasma viral suppression on clustering, a subgroup analysis was performed in a subset of participants (N = 206) with viral suppression at all visits using the same two-step clustering approach.

Demographics and HIV disease characteristics at baseline were summarized with mean (standard deviation), median (interquartile range), or N (%) and compared using a one-way analysis of variance for numeric variables or the chi-square test for categorical data between the phenotypes generated from cluster analysis. Forty-two (8.3%) participants missed the 12-month assessment, and 98 (19.4%) did not complete an 18-month assessment. Changes in GDS, BDI-II score, PAOFI total score, and IADL total score over time were analyzed using LMMs with fixed effects of phenotype, time, and their interaction, and participant-specific random intercept. Multiple comparisons were performed between Phenotype 1 (the healthiest phenotype) and other phenotypes and were adjusted using the Benjamini-Hochberg procedure. The effects of baseline medical, psychiatric, and substance use characteristics on the longitudinal BPS phenotypes were separately estimated using multinomial logistic regression in univariable analysis. Variables associated with the phenotypes at a p-value of 0.10 or less were included in the multivariable analysis. They were retained in the model as covariates if they were still associated with any one of the phenotypes with a p-value of 0.05 or less. Table 2 specifies the predictors, outcomes, and potential confounds examined. In addition, participants were classified into the four MLBPS phenotypes based on baseline BDI-II, GDS, PAOFI total, and IADL total using a random forest classification model. 10-fold cross-validation was used to evaluate the predictive model. Similarly, logistic regression was applied to classify participants as having either a healthy phenotype (P1) or the alternative (less healthy, P2–P4) in a binary fashion. Statistical analyses were performed using R version 3.6.3, 2020.

## Results

### Participants.

The 506 participants at baseline averaged in their mid-40s (mean [SD] 43.1 [8.4] years), and were mostly males (75.6%), of White or Black race (41.5% each), with some college education (12.8 [2.5] years). 30.7% were employed. The average estimated duration of HIV was 10 years, 56.5% had a prior diagnosis of AIDS, 355 (66.9%) took ART at baseline, the plasma HIV RNA was ≤ 200 copies/mL in 248 (46.7%), and nadir and current CD4 + T lymphocyte count (median [IQR]) was 173 [61, 299] and 448/μL[290, 657].

### Multidimensional longitudinal BPS phenotypes (MLBPSPs).

**Figure 1:**
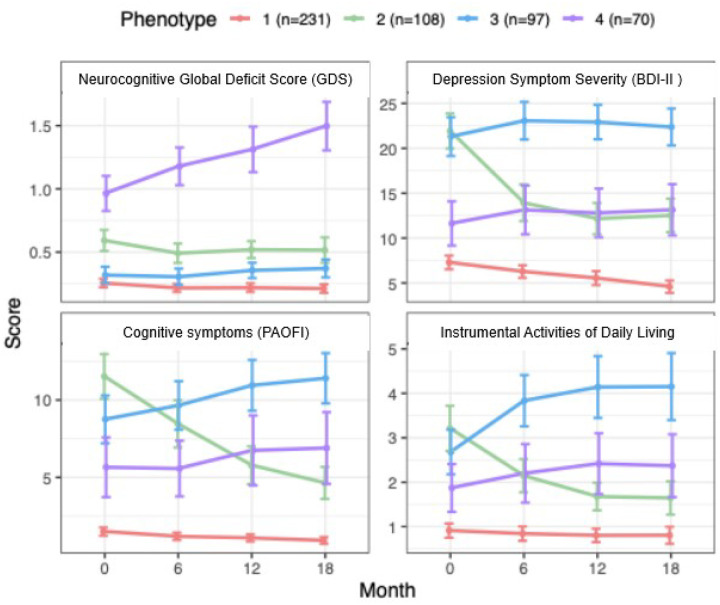
Longitudinal trajectories of neurocognitive, mood, and functional outcomes by phenotype. Higher scores on the y-axis indicate worse performance or complaints in that domain. Error bars represent 95% confidence interval. GDS, global deficit score; BDI-II, Beck Depression Inventory-II; PAOFI, Patient’s Assessment of Own Functioning Inventory; IADL = instrumental activities of daily living. P1, “stably healthy”; P2 “depressed and functionally impaired but improving”; P3 “stably depressed, disabled, spared cognition”; P4 “cognitively impaired, worsening”.

The two-step cluster analysis yielded four phenotypes based on the means and longitudinal trajectories in neurocognitive, mood, and self-reported cognitive difficulties and problems with IADLs. Figure 1 shows initial values and changes in mean GDS, BDI-II, PAOFI Total, and IADL Total over time for each phenotype. To enhance interpretability, we assigned descriptive names based on the MLBPSP feature evolution: Phenotype 1 (P1; “stably healthy”) was the largest group (N = 231; 45.6% of the total), the healthiest across all the MLBPSP features and remained stable over time. P2 (“depressed and functionally impaired but improving”; N = 108, 21.3%) had mild NCI at baseline that remained stable over time; their BDI-II scores indicated moderate depression and substantial functional disability at baseline that improved greatly during follow-up; P3 (“stably depressed, disabled, spared cognition”; N = 97; 19.2%) had minimal NCI but severe depression, cognitive symptoms, and impaired IADLs that worsened somewhat during follow-up. P4 (“cognitively impaired, worsening”) was the smallest group (N = 70, 13.8%), the most neurocognitively impaired, and had worsening neurocognitive performance over time; their depressive symptoms, cognitive symptoms, and IADL dependence were relatively mild and remained stable. **Supplementary Table 1** details baseline differences in phenotype features between the MLBPSPs.

Baseline demographic, HIV disease, and treatment characteristics by longitudinal BPS Phenotype (Tables 1 and 2). Phenotype differences in baseline age were small but significant (overall p = 0.040), with P1 (“stably healthy”) and P2 (“depressed and cognitively impaired but improving”) being the youngest (42 years), and P3 and P4 older (approximately 45). P1 had the highest proportion of males (85.3%). The ethnic distribution varied across Phenotypes, with the highest proportion of Black individuals in P2 (51.9%) and the highest proportion of Hispanic individuals in P4 (22.9%). Education differed across Phenotypes (overall p < 0.001), being highest in P1 (13.5 [SD 2.42] years). P4 had the longest duration of HIV infection, and AIDS was most prevalent in P3 and P4; none of the other HIV disease or treatment characteristics differed between the groups. Lower pre-morbid reading level (WRAT-III) was associated with baseline NCI. Quality of life (MOS-HIV) was best for P1, followed by P4, P2, and P3.

As seen in **Supplementary Fig. 1**, viral suppression rates increased on average over time for all Phenotypes. This must be considered in the context of ART guidelines used during this study at baseline when treatment was recommended only when the CD4 + T-cell count dropped below 200/μL or when a serious opportunistic condition developed. In a subgroup analysis (**Supplementary Fig. 2**), longitudinal trajectories for participants with viral suppression were similar to those for the entire group.

Table 3 shows the univariable and multivariable analyses of the associations between baseline biomedical predictors and odds ratios (ORs) for P2, P3, and P4 compared to the reference P1. In univariable analyses, several factors differentiated the phenotypes: CPD (highest OR in P4), diabetes mellitus (DM, also highest in P4), DSP (significantly more frequent in P3 and P4 than P1), DNP, polypharmacy (significantly more frequent in P2–4), Charlson Comorbidity Index (CCI; worse in P3 and P4), lifetime any substance use disorder (SUD, more in P3), and hematocrit (lower in P3). In multivariable analysis adjusting for education, the groups were differentiated by CPD (significantly more frequent in P4), lifetime any SUD (highest in P3), DNP (more frequent in P2 and P3), CCI (highest in P3), serum creatinine (lower in P3 and P4), and polypharmacy (higher in P2–4). **Supplementary Table 2** provides multivariable pairwise comparisons of baseline medical characteristics among the Phenotypes without education as a covariate.

### Secondary analysis of participants with viral suppression

In the subset of participants (N = 206) with viral suppression over all visits, the clustering analysis found longitudinal trajectories that were 80% in agreement with the clustering solution for the larger group that included both suppressed and unsuppressed participants.

### Prediction of MLBPSPs based on baseline BPS characteristics

We considered the possibility that baseline BPS characteristics alone might provide a sensitive and specific way to predict the longitudinal phenotypes. We employed a random forest classifier to categorize participants into the four MLBPSPs based on baseline neurobehavioral data, specifically the BDI-II, GDS, PAOFI total, and IADL total. The overall classification accuracy was 60.5%, with a misclassification rate of 39.5%. In a secondary analysis, the classifier’s ability to distinguish between the healthiest phenotype (P1) and the less healthy phenotypes (P2–4) using logistic regression showed an accuracy of 82.6%. This indicates a substantial predictive capability, albeit with some limitations in precise phenotype allocation, as 19.3% of participants classified into P1 were better suited for P2–4, and 15.8% of those classified into P2–4 would align more accurately with P1.

## Discussion

### Key findings

This study analyzed baseline biomedical and demographic predictors of longitudinal trajectories of neurocognitive, mood, and self-reported cognitive difficulties and problems with IADLs. Based on these features, we identified four multidimensional, longitudinal biopsychosocial phenotypes (MLBPSPs). Phenotype 1 was the largest group, the healthiest, and remained stable over time on all features. Phenotype 2 had mild, stable cognitive impairment, with substantial baseline depression and functional disability that greatly improved over the follow-up period. Phenotype 3 had minimal NCI throughout the study but substantial depressive symptoms and IADL dependence that persisted or worsened over time. Phenotype 4 was the most neurocognitively impaired and had worsening performance over time but with only mild and stable depressive symptoms and functional disability. In sum, these groups were markedly different in the nature and severity of BPS problems as well as their trajectories over time; interestingly, in both respects, depressive symptoms and functional disability tracked together (increasing or decreasing in P3 versus P2), whereas NC performance followed a different course (worsening in P4 and stable in the others). The phenotype clustering was consistent in participants with viral suppression, suggesting that the findings may generalize to modern-day cohorts. The longitudinal dissociation between neurocognitive performance and mood/functioning warrants investigations into potential differences in underlying biological mechanisms.

The healthy group (P1) showed significant but small demographic differences from the more adversely affected BPS groups (P2–4). For example, the average age in P1 was 1–2 years younger than that of P3 and P4. Their education was not significantly higher than those of the most cognitively impaired group (P4). P4 had the highest percentage of Hispanic individuals, which is consistent with prior findings of increased risk of NCI and longitudinal worsening in U.S. samples (41, 42). Quality of life tracked with the burden of depression, being best in P1 and worst in P3. Depression is a major contributor to poorer quality of life in PWH (43, 44) and PWoH (45). The “healthy” group (P1) showed significant but small demographic differences from the more adversely affected BPS groups (P2–4). For example, the average age in P1 was 1–2 years younger than that of P3 and P4. Their education was significantly higher than P2 and P3 but not significantly different than those of the most cognitively impaired group (P4). P4 had the highest percentage of Hispanic persons, which is consistent with prior findings of increased risk of cognitive impairment and longitudinal worsening in U.S. samples(46) (47) (41). Quality of life tracked with the burden of depression, being best in P1 and worst in P3. Prior publications have also shown depression to be a major contributor to poorer quality of life in PWH (48) (49) and PWoH (45).

The findings from our random forest classification analysis suggest that while baseline neurobehavioral characteristics can predict longitudinal MLBPSPs with reasonable accuracy, the complexity of individual trajectories requires a multidimensional approach for precise prognosis. The differential predictive accuracy—higher for identifying the healthiest phenotype (P1) compared to distinguishing among less healthy phenotypes (P2–4)—underscores the challenge in pinpointing specific adverse outcomes based on baseline characteristics alone. This aligns with previous research suggesting that multiple biological mechanisms may influence cognitive impairment, making it difficult to identify a concise panel of predictors. However, accurately identifying individuals likely to maintain a healthy trajectory based on the absence of negative baseline indicators remains a valuable tool in clinical settings, providing a basis for targeted interventions and monitoring.

The identified MLBPSPs bear substantial clinical relevance; some of these findings are unique and noteworthy. The healthy phenotype with stable trajectories underscores the potential to maintain a high quality of life and functional status despite the chronic nature of HIV infection. This relatively healthy group was also notable because they had the highest current employment rate. The fact that all the other middle-aged BPS groups had similarly low employment rates despite different patterns of BPS problems suggests that any of these problems (NCI, depression, or self-reported cognitive problems) may increase the risk of chronic unemployment or leaving the workforce. The healthy P1 group with the best neurobehavioral, mood, and functional outcomes also had the lowest CCI, the lowest rates of lifetime SUDs, and the least polypharmacy, CPD, DM, DNP, and DSP. Clinically, this group may require less intensive neuropsychiatric monitoring than the other BPS groups – and provides a rationale for reassurance for patients in this group concerned about NP complications.

### BPSP associations with HIV disease and treatment variables and comorbidities

This study found that baseline HIV characteristics, such as viral suppression, did not predict MLBPSPs. In contrast, comorbidities like CPD, polypharmacy, and DNP were more prevalent in less healthy BPSPs. These findings confirm and extend prior work demonstrating that comorbidity burden has become an important predictor of poor clinical evolution in PWH (50–52). The findings mirror clinical trends in HIV management, underscoring the increasing importance of managing comorbidities rather than HIV itself, which in most cases is already well-controlled by ART.

CPD’s strong link to the most adversely affected MLBPSP (P4) suggests exposure to other pulmonary risk factors. Polypharmacy’s significance across the phenotypes indicates a higher comorbidity burden or complex medical management in the less healthy phenotypes. However, polypharmacy frequently includes medications that can affect cognitive function, such as anticholinergics; therefore, deprescribing may benefit PWH (53–56). Higher DNP rates could be attributable to specific previous HIV treatments (e.g., previous exposure to neurotoxic stavudine and didanosine). Lower creatinine in the most adverse MLBPSPs may reflect loss of muscle mass, consistent with a diagnosis of sarcopenia or frailty, which was not assessed early in the CHARTER cohort. rather than better renal function. Most clinical laboratory tests did not show significant differences across the phenotypes, including aspartate transaminase levels, serum total protein, serum albumin, and serum glucose. Overall, the study highlights that adverse medical characteristics at baseline are more predictive of BPSP trajectories than demographics or HIV-specific factors, filling a gap in the literature on BPSP evolution in PWH. The pattern of comorbidities suggests that interventions for CPD, polypharmacy, and DNP may improve long-term outcomes.

P2 exhibited significant improvement over time despite initial depression and disability, suggesting resilience and potential benefits from targeted mental health services and rehabilitation programs. The management of P3, the group with persistent moderate depression and perceived disability but relatively preserved neurocognitive performance, may be particularly challenging. This group had numerous problems at baseline, including high average CCI value, polypharmacy, DSP, and DNP, and the highest frequency of lifetime SUDs. Participants in this group may require a more intensive approach that addresses their medical comorbidities, mental health, and functional status, possibly involving multidisciplinary care with physicians, mental health professionals, occupational therapists, and social workers.

P4, the most neurocognitively impaired group with worsening cognition, appears particularly vulnerable. They had the longest duration of HIV infection, high CCI values, the most CPD, a relatively high prevalence of DM and DSP, and the most polypharmacy. On the other hand, their comorbidities and worsening cognition occurred within the context of less indication of mental health disturbances (depressive symptoms or SUD). Clinical management of these individuals is likely to be complex, necessitating closer neurocognitive monitoring, mitigation of neuromedical risks, mental health support, and balancing reducing polypharmacy with the possible addition of pharmacological treatment to address cognitive decline. This group also highlights the need for ongoing research into neuroprotective strategies and the management of comorbid conditions that may drive or exacerbate neurocognitive decline.

### Overlap between the MLBPSPs

Some individuals in each phenotype had characteristics that fell outside the typical range for the designated phenotype. Also, the characteristics of these individuals in one or more domains were similar to the distribution of other phenotypes. This overlap in characteristics across phenotypes might be attributable to several factors related to the methods used and inherent to the individuals studied. In the context of HIV and its impact on neurocognitive, mood, and functional domains, individual variability is expected. Even within a healthy phenotype, some individuals may have exhibited adverse trajectories in one or two BPS domains due to many factors. These could include genetic predispositions, the presence of comorbid conditions, viral factors, differences in treatment responses, or lifestyle factors that were not captured fully by our assessment or the clustering analysis. Resilience factors, such as strong social support networks, effective coping strategies, and positive health behaviors, might protect individuals from adverse outcomes in other domains, even if they exhibit challenges in specific areas. For instance, an individual in the stably healthy phenotype with a somewhat negative trajectory in a particular BPS domain might maintain overall health due to better overall resilience.

The longitudinal nature of the study adds another layer of complexity. Changes over time in health status, treatment efficacy, and personal circumstances can lead to shifts in health trajectory, which the analysis may not fully capture at every point in time. Furthermore, measurement error and variability in the assessment tools used to evaluate the BPS domains could contribute to overlap. For instance, self-report measures can be subjective and influenced by current mood or recent experiences, which may not accurately reflect long-term trends. Future studies could benefit from incorporating a more nuanced understanding of individual variability and resilience factors, possibly through mixed-methods approaches that combine quantitative analysis with qualitative insights. The clustering algorithms in machine learning are probabilistic and rely on optimizing certain criteria or distance measures. They do not always yield completely distinct clusters, particularly when applied to complex human data that may not be linearly separable or may have underlying structures that are not easily discernible by the algorithm. This can lead individuals with outlier trajectories to be grouped with clusters that do not entirely match their profiles.

### Strengths

This study has several notable strengths that contribute to its scientific merit and potential impact on neurobehavioral research in HIV. One of these is the recruitment of a demographically diverse participant pool that mirrors the broader U.S. population of people with HIV. This enhances the study’s external validity, allowing for more generalizable findings across various subsets of the PWH population. Our longitudinal design allows for the observation of trends and patterns over time, providing valuable insights into the progression of neurobehavioral decline and the potential long-term effects of HIV on brain function. The application of sophisticated statistical techniques exemplifies a rigorous approach to data analysis and the ability to detect subtle impairments across multiple domains, providing a nuanced understanding of NCI among PWH. The study used a comprehensive BPS assessment protocol using high-quality, validated instruments. The operationalization of NCI using a GDS threshold provides a clear and quantifiable criterion for identifying cognitive deficits, which aids in standardizing research outcomes and facilitating comparisons with other studies. By converting raw scores into demographically corrected standardized scores, the study accounts for potentially confounding variables such as age, education, sex, ethnicity, and race and ethnicity. These strengths collectively underscore the study’s potential to inform clinical practice, guide future research, and influence policy-making in the realm of HIV-related cognitive health.

### Limitations

Despite our robust methods, their limitations should be considered. Compared to contemporary cohorts, in which viral suppression rates are very high, this cohort’s suppression rate was approximately 70% at follow-up. As previously noted, this should be considered in the context of ART guidelines in effect at the time of the study (57). The U.S. Department of Health and Human Services updated its guidelines in 2015 to recommend ART for all PWH, regardless of CD4 + T-cell count. Additionally, while our selection criteria aimed to create a sample representative of the population of adults with HIV in the U.S., the exclusion of individuals with active, uncontrolled conditions and ongoing SUDs may limit the generalizability of our results. Determining “active, uncontrolled” conditions was based on clinical assessments at the time of enrollment, which may not capture the dynamic nature of these conditions. Future studies should consider longitudinal tracking to observe how fluctuations in comorbid conditions influence BPSP features. While we employed standard and advanced statistical methods to manage the complexity of our data, not all potential confounders were addressed, which may affect our results. Future analyses should incorporate additional variables such as social determinants of health(18), ART adherence, and genetic factors that may contribute to NCI. Women were underrepresented in the cohort, although our numbers approximate the proportion of women with HIV in the U.S. We did not have data available on people without HIV for comparison to PWH. Such a comparison might provide unique insights into the factors underlying BPS manifestations in PWH compared to the remainder of the population.

These limitations notwithstanding, our findings have significant potential implications for clinical practice. Even when viral suppression was achieved, NCI and functional impairment persisted, further underscoring the need for continued work in this area to develop clinical tools for screening and classification to enable early interventions in PWH most at risk of future decline. Thus, routine screening could be integrated into standard HIV care to identify individuals at risk for NCI early when interventions may be most successful. Moreover, our results provide a foundation for developing targeted cognitive rehabilitation programs and inform policymakers about resource allocation for this purpose. This study contributes to understanding the complex interplay between HIV infection, NC performance, mood, and daily functioning. Future research could focus on better understanding the underlying mechanisms driving these different phenotypes - including inflammation, immune activation, oxidative stress, and vascular risk. Identifying distinct phenotypes opens avenues for personalized approaches to care and interventions for PWH living with these disorders. Tailoring interventions to a phenotype might involve combining specific antidepressant medications and neuroprotective agents or minimizing the use of medications that contribute to NCI (e.g., anticholinergics). Pursuing these findings could significantly enhance PWH’s daily functioning and quality of life.

## Figures and Tables

**Figure 2:**
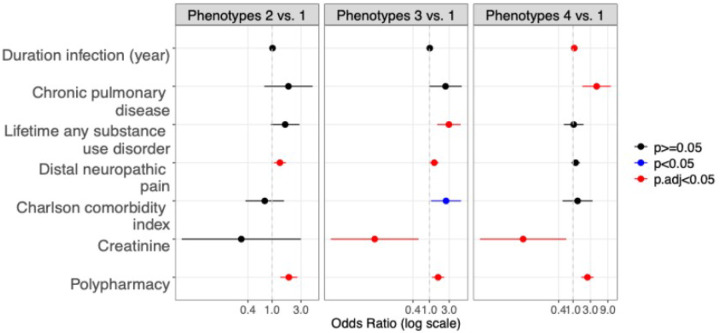
Associations between medical variables and the MLBPS phenotypes in multivariable analysis using multinomial logistic regression. Odds ratio ranges were calculated relative to Phenotype 1. Error bar represents 95% confidence interval. Red color denotes *p*<0.05 after multiple comparison correction using the Benjamini Hochberg (BH) procedure and blue denotes p ≥ 0.05 after the BH correction; Polypharmacy= number of concomitant non-antiretroviral medications; p.adj= adjusted p-value with the BH procedure.

**Table 1:** Baseline demographic, HIV disease, and antiretroviral treatment characteristics by phenotype

	Phenotype 1 (n = 231)	Phenotype 2 (n = 108)	Phenotype 3 (n = 97)	Phenotype 4 (n = 70)	Overall p	Pairwise Comparisons^c^
Demographics						
Age (year)	42.6 (8.18)	42.5 (8.5)	45.0 (8.76)	45.8 (7.33)	**.005**	1, 2 < 3, 4^d^
Education (year)	13.5 (2.42)	12.6 (2.87)	12.5 (2.46)	13.0 (2.63)	**.002**	1 > 2, 3
Male	197 (85.3%)	77 (71.3%)	73 (75.3%)	54 (77.1%)	**.013**	1 > 2
Ethnicity					**.013**	
Black	106 (46.1%)	56 (51.9%)	38 (39.2%)	20 (28.6%)		
Hispanic	22 (9.57%)	9 (8.33%)	9 (9.28%)	16 (22.9%)		
White	99 (43.0%)	42 (38.9%)	48 (49.5%)	31 (44.3%)		
Other	3 (1.3%)	1 (0.93%)	2 (2.06%)	3 (4.29%)		
WRAT-III reading score	97.4 (13.7)	83.8 (16.7)	97.5 (11.8)	89.9 (15.9)	**< .001**	1, 3 > 2,4^d^
Employment	103 (44.6%)	24 (22.2%)	21 (21.7%)	15 (21.4%)	**< .001**	1 > 2, 3,4
HIV Disease Characteristics						
Duration of ARVs (months)^a^	55.9 (50.1)	62.5 (57.2)	56.3 (43.6)	84.1 (58.1)	**.003**	4 > 1, 2,3^d^
Duration of HIV (year)^a^	8.82 (6.41)	9.96 (6.4)	10.9 (6.35)	12.4 (5.74)	**< .001**	4 > 1,2; 3 > 1^d^
AIDS diagnosis	122 (52.8%)	62 (57.4%)	69 (71.1%)	47 (67.1%)	**.009**	3 > 1^d^
Current CD4 T-Cells (/μL)^a^	434 [295, 666]	460 [313, 601]	491[289, 677]	436 [239, 628]	.86	
Nadir CD4 + T- Cells (/μL)^a^	193 [30, 359]	179 [48, 300]	160 [63, 286]	110 [33, 215]	.19	
On ART	156 (67.5%)	73 (67.6%)	71 (73.2%)	55 (78.6%)	.27	
Plasma HIV RNA ≤ 200 c/mL	109 (48.4%)	47 (43.9%)	53 (55.2%)	39 (56.5%)	.26	
CSF HIV RNA ≤ 200 c/mL	151 (73.0%)	72 (75.8%)	55 (64.7%)	53 (80.3%)	.16	
CNS penetration effectiveness rating	7.99 (2.08)	8.3 (1.91)	8.23 (2.21)	8.22 (1.82)	.69	

Notes: Significant variables **bolded**:

a square root transformations before comparative analysis, raw values reported in the table; WRAT = wide range achievement test; Values are mean (standard deviation) or number of cases (%);

c Three pairs comparisons (2, 3, and 4 vs. 1, where 1 = Phenotype 1; 2 = Phenotype 2; 3 = Phenotype 3; 4 = Phenotype 4) were performed and adjusted using the Benjamini Hochberg procedure for multiple comparisons (k = 3);

d Six pairs comparisons between Phenotypes 1–4 (k = 6).

**Table 2:** Baseline biomedical, psychiatric, and substance use characteristics by Phenotype.

Characteristics	Phenotype 1 (n = 231)	Phenotype 2 (n = 108)	Phenotype 3 (n = 97)	Phenotype 4 (n = 70)	Overall P	Pairwise Comparisons^c^
*Medical Comorbidities*						
Diabetes mellitus	17 (7.36%)	11 (10.19%)	9 (9.28%)	12 (17.14%)	.12	
Hypertension	44 (19.05%)	16 (14.81%)	15 (15.46%)	18 (25.71%)	.26	
Hyperlipidemia	22 (9.52%)	15 (13.89%)	11 (11.34%)	8 (11.43%)	.69	
Chronic pulmonary disease	13 (5.63%)	9 (8.33%)	11 (11.34%)	14 (20%)	**.003**	4 > 1
Major neurocognitive comorbidity	11 (4.76%)	17 (15.74%)	9 (9.28%)	24 (34.29%)	**< .001**	2, 4 > 1
DSP Signs (> = 2)	52 (31.52%)	31 (38.75%)	40 (57.97%)	28 (57.14%)	**< .001**	3, 4 > 1
Number of non-antiretroviral medications (median (IQR))	1 (0, 2)	2 (1, 4)	2 (1, 4)	3 (1, 4.75)	**< .001**	2, 3, 4 > 1
Total d-drug exposure (months)^a^,	0 [0, 36.0]	0 [0, 33.9]	12.5 [0, 36]	5.42 [0, 52.4]	0.47	
On tenofovir	97 (41.99%)	46 (42.59%)	41 (42.27%)	34 (48.57%)	0.8	
MOS HIV quality of life					**< .001**	
= 0	1 (0.43%)	2 (1.85%)	2 (2.08%)	1 (1.43%)		
= 25	5 (2.17%)	9 (8.33%)	15 (15.62%)	6 (8.57%)		
= 50	50 (21.74%)	54 (50%)	54 (56.25%)	21 (30%)		
= 75	119 (51.74%)	33 (30.56%)	19 (19.79%)	32 (45.71%)		
= 100	55 (23.91%)	10 (9.26%)	6 (6.25%)	10 (14.29%)		
*Clinical Chemistry*						
Aspartate transaminase^b^ (IU/L)	39.69 (32.23)	36.29 (17.37)	37.05 (30.05)	41.58 (27.53)	.45	
Serum total protein (g/dL)	7.95 (0.85)	7.99 (0.86)	7.81 (0.82)	7.86 (1.02)	.42	
Serum albumin (g/dL)	4.26 (0.44)	4.22 (0.41)	4.2 (0.38)	4.22 (0.51)	.64	
Hematocrit (%)	41.66 (4.36)	41.14 (4.23)	40.6 (4.12)	41.22 (4.98)	.24	
Creatinine^b,c^	0.97 (0.23)	0.95 (0.27)	0.92 (0.18)	0.94 (0.32)	.20	
*Psychiatric and Substance Use*						
BDI-II Total score^a^	7.68 (6.07)	18.04(9.98)	25.5 (10.25)	9.99 (8.58)	**< .001**	2, 3, 4 > 1
Lifetime MDD	92 (38.7%)	46 (57.5%)	90 (78.3%)	35 (48.0%)	**< .001**	3, 4 > 1
Lifetime alcohol use disorder	115 (48.3%)	43 (53.8%)	80 (69.6%)	38 (52.1%)	**.002**	3 > 1
Lifetime cannabis use disorder	67 (28.2%)	25 (31.3%)	35 (30.4%)	21 (28.8%)	.94	
Lifetime Meth use disorder	38 (16.0%)	12 (15.0%)	22 (19.1%)	9 (12.3%)	.65	
Lifetime Any substance use disorder	157 (66.0%)	61 (76.3%)	97 (84.4%)	47 (64.4%)	**.001**	3 > 1
Cumulative density nicotine^a^	13 (9)	16 (12)	14 (10)	16 (11)	.099	2 > 1

Note:

^a^square root and ^b^log10 transformations before comparative analysis;

c the subjects with creatinine greater than 6 were removed form analysis (n = 6); BDI-II = beck depression inventory-II; DSP = distal sensory polyneuropathy; IU = international units; MDD = major depressive disorder; PAOFI = assessment of own functioning inventory; WRAT = wide range achievement test; Values are mean (standard deviation) or number of cases (%);

c Three pairs comparisons (2, 3, and 4 vs. 1, where 1 = Phenotype 1; 2 = Phenotype 2; 3 = Phenotype 3; 4 = Phenotype 4) were performed and adjusted using the Benjamini Hochberg (BH) procedure for multiple comparisons (k = 3);

d Adding comparisons between Phenotypes 2, 3, 4 (k = 6).

**Table 3:** Univariable and multivariable pairwise comparisons of baseline medical characteristics among the BPS Phenotypes (P1–4). Odds ratios represent pairwise comparisons between P1 (the reference group) and each of the other Phenotypes. Significant values are **bolded**.

Predictor	P2 vs P1		P3 vs P1		P4 vs P1	
	OR (95% CI)	p-value	OR (95% CI)	p-value	OR (95% CI)	p-value
**Univariable analysis**						
**CPD**	1.52 (0.63, 3.68)	0.35	2.14 (0.93, 4.97)	0.075	**4.19 (1.86, 9.42)**	**0.001**
**Diabetes mellitus**	1.43 (0.64, 3.16)	0.38	1.29 (0.55, 3.00)	0.56	**2.60 (1.18, 5.76)**	**0.018**
Hypertension	0.74 (0.40, 1.38)	0.34	0.78 (0.41, 1.48)	0.44	1.47 (0.78, 2.76)	0.23
Hyperlipidemia	1.53 (0.76, 3.09)	0.23	1.22 (0.56, 2.61)	0.62	1.23 (0.52, 2.89)	0.64
**DSP**	1.37 (0.79, 2.40)	0.26	**3.00 (1.68, 5.35)**	**< 0.001**	**2.90 (1.51, 5.57)**	**0.001**
**DNP**	**1.50 (1.22, 1.85)**	**< 0.001**	**1.52 (1.22, 1.88)**	**< 0.001**	**1.43 (1.13, 1.83)**	**0.003**
**Polypharmacy**	**2.10 (1.56, 2.84)**	**< 0.001**	**1.91 (1.40, 2.59)**	**< 0.001**	**2.58 (1.82, 3.66)**	**< 0.001**
**CCI**	1.47 (0.80, 2.71)	0.22	**4.09 (1.98, 8.44)**	**< 0.001**	**3.53 (1.58, 7.91)**	**0.002**
LT alcohol use disorder	1.31 (0.82, 2.07)	0.26	**2.12 (1.29, 3.48)**	**0.003**	1.24 (0.73, 2.12)	0.43
LT cannabis use disorder	1.34 (0.81, 2.21)	0.25	1.44 (0.86, 2.41)	0.17	1.43 (0.80, 2.55)	0.23
LT meth use disorder	1.36 (0.74, 2.51)	0.32	1.56 (0.84, 2.88)	0.16	0.77 (0.34, 1.76)	0.54
**LT any SUD**	**1.83 (1.08, 3.08)**	**0.024**	**3.26 (1.74, 6.11)**	**< 0.001**	1.12 (0.64, 1.98)	0.68
Serum glucose^a^ (mg/dL)	4.58 (0.84, 24.9)	0.078	2.37 (0.39, 14.3)	0.35	4.45 (0.63, 31.5)	0.14
**Hematocrit (%)**	0.97 (0.92, 1.03)	0.31	**0.95 (0.90, 1.00)**	**0.046**	0.98 (0.92, 1.04)	0.46
Serum albumin (g/dL)	0.80 (0.47, 1.37)	0.42	0.72 (0.42, 1.25)	0.25	0.78 (0.42, 1.46)	0.44
Total serum protein (g/dL)	1.05 (0.80, 1.36)	0.74	0.82 (0.62, 1.09)	0.17	0.89 (0.65, 1.21)	0.46
Creatinine^a^ (mg/dL)	0.25 (0.027, 2.38)	0.23	0.13 (0.012, 1.34)	0.087	0.11 (0.008, 1.60)	0.11
AIDS status	1.20 (0.76, 1.91)	0.43	**2.20 (1.32, 3.66)**	**0.002**	1.83 (1.04, 3.20)	0.036
Duration of HIV infection (yr)	1.03 (0.99, 1.07)	0.12	**1.05 (1.01, 1.09)**	**0.008**	**1.10 (1.05, 1.15)**	**< 0.001**
**Multivariable analysis**						
**Education (year)**	**0.87 (0.78, 0.96)**	**0.005**	**0.88 (0.80,0.98)**	**0.02**	0.92(0.82, 1.03)	0.16
**Duration of HIV infection (yr)**	1.01 (0.97, 1.06)	0.56	1.02 (0.97, 1.06)	0.47	**1.07 (1.02, 1.13)**	**0.007**
**CPD**	1.70 (0.67, 4.31)	0.26	2.23 (0.89, 5.56)	0.086	**4.18 (1.68, 10.4)**	**0.002**
**LT any SUD**	1.35 (0.77, 2.36)	0.3	**2.52 (1.30, 4.88)**	**0.006**	0.92 (0.48, 1.76)	0.81
**DNP**	**1.35 (1.08, 1.70)**	**0.01**	**1.32 (1.04, 1.66)**	**0.021**	1.18 (0.90, 1.54)	0.23
**CCI**	0.72 (0.34, 1.50)	0.38	**2.42 (1.05, 5.61)**	**0.039**	1.28 (0.49, 3.31)	0.62
**Creatinine**^a^ **(mg/dL)**	0.40 (0.042, 3.92)	0.43	**0.059 (0.005, 0.68)**	**0.023**	**0.049 (0.003, 0.76)**	**0.031**
**Polypharmacy** ^b^	**1.98 (1.43, 2.76)**	**< 0.001**	**1.68 (1.20, 2.36)**	**0.003**	**2.55 (1.72, 3.77)**	**< 0.001**

Notes: ^a^ log10 and ^b^ square root transformation prior to analysis; bold values represent significance at the p < 0.05 after multiple comparison correction with the BH method (k = 3); P1 = Phenotype 1; P2 = Phenotype 2; P3 = Phenotype 3; P4 = Phenotype 4; LT = lifetime; meth = Methamphetamine; polypharmacy = number of concomitant non-antiretroviral medications. SUD = substance use disorder. DSP = Distal sensory polyneuropathy. DNP = Distal neuropathic pain. CCI = Charlson comorbidity index.

## Data Availability

Upon publication of this manuscript, de-identified data and data dictionaries will be available to interested investigators through the National NeuroAIDS Tissue Network (NNTC).Requests for the data will be submitted through their website (https://nntc.org). Data and data dictionaries will be provided to qualified investigators upon approval of their request and with a signed data use agreement.

## Research in Context

Neuropsychiatric complications such as neurocognitive impairment (NCI) and depression are common in people with HIV (PWH) despite viral suppression on antiretroviral therapy (ART), but these conditions are heterogeneous in their clinical presentations and associated disability. We searched the PubMed database from 2010–2023 for publications using search terms including HIV, neurocognitive, and phenotype; these are appropriately referenced in the manuscript. As no clinical trials have been performed in this area, all evidence is observational. The majority of studies looked at cognitive phenotypes only and the clustering methods ranged from latent profile analysis to machine learning. However, only limited longitudinal work has been published in this area, and the duration of follow-up in those studies is, on average 6 months. Therefore, we sought to evaluate the connection between biopsychosocial phenotypes and medical characteristics over time. Our findings extend and enhance the existing literature by combining cognition with mood, activities of daily living, and cognitive complaints to generate complex biopsychosocial phenotypes and assessing their relationship for clinical variables over 18 months. Negative longitudinal phenotype trajectories were associated with chronic pulmonary disease, distal neuropathic pain and creatinine levels. Together with previously published work, these findings have significant implications for HIV care by identifying PWH with clinical characteristics that predict future adverse trajectories.
